# Handheld Capillary Blood Lactate Analyzer as an Accessible and Cost-Effective Prognostic Tool for the Assessment of Death and Heart Failure Occurrence during Long-Term Follow-Up

**DOI:** 10.1155/2016/5965782

**Published:** 2016-12-28

**Authors:** Grzegorz M. Kubiak, Wojciech Jacheć, Celina Wojciechowska, Magdalena Traczewska, Agnieszka Kolaszko, Leszek Kubiak, Joanna Jojko, Ewa Nowalany-Kozielska

**Affiliations:** ^1^Clinical Department of Cardiovascular Diseases, Multidisciplinary Tertiary Hospital, Medical University of Silesia, 10 Curie-Skłodowska Street, 41-800 Zabrze, Poland; ^2^Internal Medicine Department, Pyskowice Municipal Hospital, 2 Szpitalna Street, 44-120 Pyskowice, Poland; ^3^2nd Department of Cardiology in Zabrze, Medical University of Silesia, Katowice, Poland

## Abstract

Impact of tissue lactate accumulation on prognosis after acute myocardial infarction (AMI) is biased. The study aimed to assess the prognostic role of lactate concentration (LC) in patients with AMI during one year of follow-up. 145 consecutive patients admitted due to AMI were enrolled. The data on the frequency of endpoint occurrence (defined as I, death; II, heart failure (HF); and III, recurrent myocardial infarction (re-MI)) were collected. The patients were divided into group A (LC below the cut-off value) and group B (LC above the cut-off value) for the endpoints according to receiver operating characteristic (ROC) analysis. The cumulative survival rate was 99% in group I-A and 85% in group I-B (*p* = 0.0004, log-rank test). The HF-free survival rate was 95% in group II-A and 82% in group II-B (*p* = 0.0095, log-rank test). The re-MI-free survival rate did not differ between groups. A multivariate Cox analysis showed a statistically significant influence of LC on death [Hazard Ratio (HR): 1.41, 95% Confidence Interval (CI) (1.13–1.76), and* p* = 0.002] and HF [HR: 1.21, 95% CI (1.05–1.4), and* p* = 0.007] with no impact on re-MI occurrence. LC in capillary blood may be considered a useful prognostic marker of late-onset heart failure and death after AMI.

## 1. Introduction

Cardiovascular diseases are the most common cause of death in Poland, with an annual death rate of 452 per 100,000. Cardiovascular diseases thus represent a serious health problem for the country [1]. The morbidity rate of acute myocardial infarction (AMI), as shown by Yeh et al. [2], showed a tendency to decrease systematically. Irrespective of a broad spectrum of activities, a further decrease in the mortality rate primarily depends on innovation in pharmacotherapy and primary percutaneous coronary intervention (PCI) delay shortening. The abovementioned actions may positively impact the AMI in-hospital mortality rates being reported in Europe as 7% in the STEMI and 3–5% in the NSTEMI group of patients [3–6]. Patient-dependent delay plays a crucial role in the potential improvement of AMI treatment results; there is thus a need for the implementation of an easy-to-use and cost-effective diagnostic and prognostic tool. AMI is associated with an anaerobic switch phenomenon, which is primarily due to coronary artery occlusion, leading to the acute impairment of myocardium oxygen supply. A deterioration in left ventricle contractility leading to a reduction in cardiac output may result in peripheral tissue hypoxemia and inhibit glycolysis, which represents the primary source of adenosine triphosphate (ATP) supply, providing thirty-eight moles of ATP from one molecule of glucose. Pyruvate reduction into lactate restores only two moles of ATP in anaerobic conditions and is thus largely ineffective. Lactate was discovered by Scheele [7] in 1780 and was implemented into a clinical setting more than sixty years later by Johann Scherer [8] for various methods of assessment including a capillary blood handheld analyzer. The accumulation of lactate increases tissue acidosis with subsequent acid-base homeostasis disturbance, resulting in extreme conditions and symptoms of shock (tachycardia, tachypnea, cyanosis, pallor, third heart sound, and cold extremities) [9]. Whether the type of shock (hypovolemic, septic, or cardiogenic) differentially impacts the lactate concentration (LC) remains to be addressed with appropriate randomized multicenter studies.

## 2. Aim of the Study

Aim of the study was to assess LC in AMI patients; to evaluate the potential relationships between LC and other clinical and biochemical factors; to assess the possible prognostic impact of LC on death, heart failure (HF), and recurrent myocardial infarction (Re-MI) occurrence in one year of follow-up.

## 3. Methodology and Patients

We enrolled 145 consecutive patients into a prospective cohort study. They were admitted to our center (a tertiary university hospital) due to AMI between August and December 2012 and in them we performed LC measurement using the handheld The Edge® device (provided by the APEXBIO Company, Taiwan). The total cost of the equipment, including the electronic analyzer device and the measurement strips, was 410 USD, which equals 2.83 USD per patient. The study complies with the Declaration of Helsinki and was approved by the local ethics committee (approval number KNW/0022/KB1/99/12), and all patients gave informed consent prior to enrollment. The inclusion criteria included AMI according to the current European Society of Cardiology (ESC) definition [4] and age between 18 and 80 years. All enrolled patients were submitted for invasive coronary angiography. The exclusion criteria were a lack of informed consent (unconscious patients) or known malignant disease. Patients received typical treatment according to the current ESC guidelines, which were independent of the results of the LC measurement and were submitted for revascularization via percutaneous coronary intervention (PCI) coronary artery bypass grafts (CABG) or optimal medical therapy (OMT) either ad hoc or after a heart team decision process. The enrolled patients were subsequently analyzed according to the defined endpoints, which included death, HF, and re-MI occurrence in one year of follow-up performed in the majority of cases during clinical examination (90%) or telephone survey (10%). Nevertheless each endpoint which occurred during follow-up had to be confirmed by the data received from the national health care system digital database. The implemented system of blinded control during follow-up tended to improve the objectivity of the acquired data. The patients characteristics divided into STEMI and NSTEMI group were presented in the Table 1.

## 4. Statistical Analysis

The distributions of the examined parameters were analyzed using the Shapiro-Wilk test. Values were presented as the means and standard deviation (SD) or as the median in the 25th and 75th percentiles. Nominal and categorical values were expressed in percentages or proportional rates. Linear variables with a normal distribution were compared using Student's *t*-test. Variables with an abnormal distribution were compared using the Kolmogorov-Smirnov and Mann–Whitney *U* tests. Categorical variables of abnormal distribution were compared using a Chi-square test with Yates correction. A Kaplan-Meier analysis (log-rank test) was used to demonstrate the frequency of endpoint occurrence during the follow-up period for the patients, which were divided into two groups with low (A) and high (B) LC according to the calculated cut-off value (acquired from receiver operating characteristic (ROC) analysis). Cut-off values for each endpoint were calculated using ROC with subsequent sensitivity and specificity, area under curve (AUC), and Confidence Interval estimation. Cox proportional hazard regression was used to evaluate the risk of endpoint occurrence. The independent variables included in the multivariate model were variables that reached statistical significance (set at 0.1) in the univariate analysis of all parameters. Independent predictors of endpoint occurrence were presented as the Hazard Ratio (HR) with a Confidence Interval (CI). Differences between the values were considered statistically significant if *p* < 0.05. Analyses were performed using Statistica 10 with the medical package (StatSoft Inc.).

## 5. Results

STEMI patients are characterized by the more frequent use of GPIIb/IIIa receptor blockers (48 versus 9%, *p* < 0.001), lower baseline left ventricular ejection fraction (LVEF) (42 versus 49%, *p* < 0.005), and shorter time from the onset of symptoms to first medical contact (FMC) (8 versus 22 hours, *p* < 0.001). Patient characteristics according to AMI type are presented in Table 1. STEMI patients had a higher serum blood concentration of glucose (8.4 versus 7.3 mmol/l, *p* < 0.001) and white blood cell count (12 versus 10*∗*10^3/ul, *p* < 0.025). Data are presented in Table 2.

The highest AUC of 0.9 (with a specificity and sensitivity of 80% at the LC cut-off value of 5.35 mmol/l) was observed for the death endpoint; this was marked as I. All data are presented in Table 3

**Figure 1:**
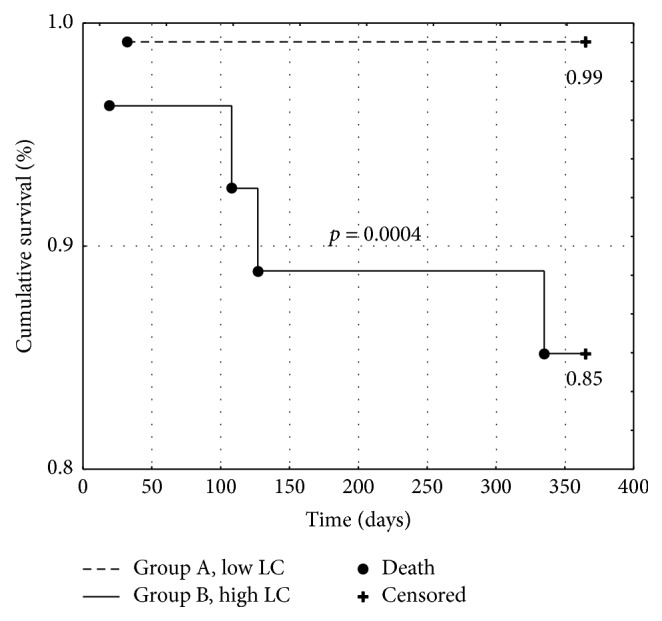
Kaplan-Meier survival curves for all-cause mortality of patients with low (group A) versus high (group B) lactate concentration. LC: lactate concentration.

**Figure 2:**
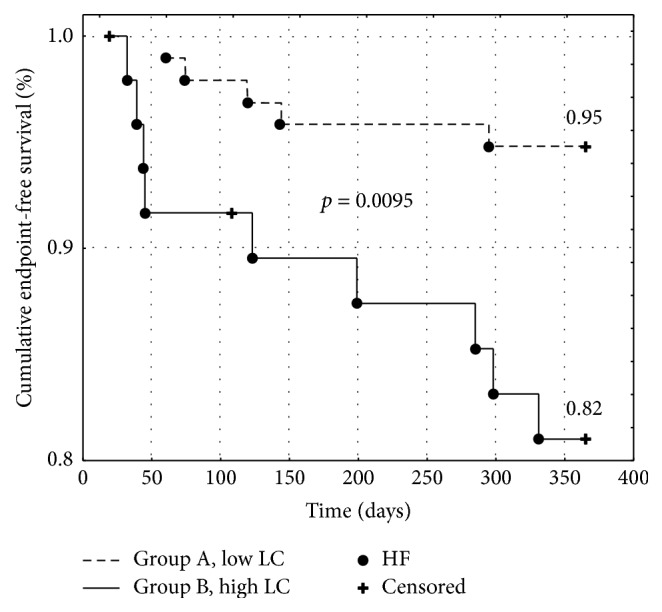
Kaplan-Meier event-free survival curves for heart failure occurrence in patients with low (group A) versus high (group B) lactate concentration. HF: heart failure. LC: lactate concentration.

**Figure 3:**
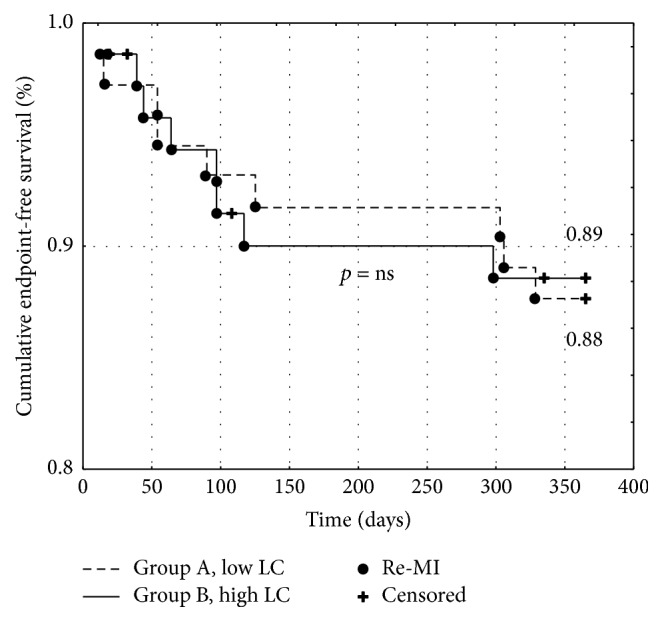
Kaplan-Meier event-free survival curves for recurrent myocardial infarction occurrence in patients with low (group A) versus high (group B) lactate concentration. LC: lactate concentration. Re-MI: recurrent myocardial infarction.

Kaplan-Meier curves were used to assess the cumulative event-free survival for each endpoint. Patients with an LC below the cut-off value had a significantly higher cumulative survival rate (0.99 versus 0.85, *p* < 0.001, Figure 1) and HF-free episode survival rate (0.95 versus 0.82, *p* < 0.01, Figure 2) with no differences in the re-MI episode survival rate (0.89 versus 0.88, *p* = ns, Figure 3).

We performed a univariate Cox regression analysis; we then constructed multivariate Cox regression analysis models using the backward stepwise method to predict the occurrence of each endpoint (I, death; II, HF; III, re-MI). A statistically significant influence of LC on death [Hazard Ratio (HR): 1.41, 95% Confidence Interval (CI) (1.13–1.76), and *p* = 0.002] and HF occurrence [HR: 1.21, 95% CI (1.05–1.4), and *p* = 0.007] was reported. The relationship between LC and re-MI during follow-up was not observed. The Cox regression analysis models are presented in Tables 4–6.

## 6. Discussion

The blood acquisition site substantially impacts LC. What should be considered a significant elevation of LC is in this condition questioned. In our study, a significant elevation was described by the production of more than 2.5 mmol/l; however, LC elevation and its role in the diagnosis of certain clinical states (in addition to prognosis and survival) have not been thoroughly elucidated. Boldt et al. [10] reported the inability to discriminate serious significant differences between LC as assessed using the handheld Accusport diagnostic tool by Roche Diagnostics in addition to the Chiron Diagnostics 865 and Lactate PAP tools by Analyticon; however, the initial incoherence between the arterial and capillary blood LC assessments were potentially due to technical (different volumes submitted to examination and method of acquisition) and clinical reasons including reduced intravascular osmotic pressure and capillary edema caused by intravenous liquid administration. The delay in LC assessments using the reference method varied between 45 and 168 minutes (mean of 85 minutes) and was twice as expensive as the electronic analyzer with a total cost of five hundred United States dollars (USD). A tight correlation between fingertip and whole blood point-of-care (POC) LC assessment in emergency department patients compared with a standard laboratory analyzer with intraclass correlation coefficients of 0.90 and 0.92, respectively, was reported by Gaieski et al. [11]. Notably, the time of LC assessment using an electronic analyzer compared with a reference method was 65 minutes shorter in this setting. The impact of LC on infection frequency was previously reported by Claridge et al. [12] and his group in patients hospitalized in the intensive care unit (ICU) after trauma or surgical intervention. In the study, the normalization of LC in time corresponded to the infection occurrence rate (a longer period of time was needed to normalize the higher incidence of infection). The mortality rate was higher in patients with the infection (7.9 versus 1.9%, *p* < 0.05) and was indirect proof of LC utility in the early risk assessment and determination of prognosis. Notably, the group of patients with an LC above 2.4 mmol/L was characterized by a higher rate of death, longer hospital stay, and increased cost of hospitalization. These findings are in accordance with our results, although we examined different group of patients with AMI submitted for invasive coronary angiography and followed up for one year. We believe that this novel approach might be useful in early risk stratification, especially considering low cost of the single LC assessment and its accessibility, although further studies in larger groups of patients and different clinical scenarios would improve the knowledge in the field. Meregalli et al. [13] reported the role of LC in the prognosis of patients after surgical treatment followed by admittance to the ICU. They demonstrated its effectiveness in assessing the risk of death and performed serious complications risk stratification with the area under ROC curves only slightly lower than those for the widely used SAPS II (New Simplified Acute Physiology Scale) [0.583 versus 0.705 (for death); 0.646 versus 0.678 (for serious complications)]. LC was thus postulated to be more reliable than other indices of hemodynamic state deterioration, such as heart rate, diuresis and the mean arterial pressure, or metabolic acidosis. The elimination of the clinical signs of shock does not exclude the possibility of hypoperfusion as an important risk factor of serious complications. It is worth mentioning that, after excluding patients with the clinical signs of shock, LC was significantly higher in those who reached endpoints I (8.7 versus 3.8, *p* = 0.008) and II (5.0 versus 3.8, *p* = 0.028). This relationship did not occur for endpoint III (3.7 versus 4.0, *p* = ns). Howell et al. [14] reported that increased LC (≥4.0 mmol/l) had serious implications for the mortality rate even in normotensive patients. The mortality rate in this group of patients was 15%; however, the patients presenting with septic shock or an LC above 4.0 mmol/l had a mortality rate of 28.3%, which was significantly higher than that in patients who had neither (2.5%, *p* < 0.0001). In a model with good discrimination (AUC = 0.87) consisting of age, blood pressure, malignancy, platelet count, and blood urea nitrogen, LC was considered to be associated with mortality. These findings were also consistent with our results despite the fact that we focused on the potential relationship between LC evaluated during AMI and symptoms of late-onset. The construction of a Cox proportional hazard regression models required the implementation of advanced statistical methods. The ROC-derived cut-off values for each of the three analyzed endpoints varied between 3.3 and 5.4 mmol/l. Jansen et al. [15] analyzed over one hundred patients and reported an increased mortality rate in those with an LC above 3.5 mmol/l (41 versus 12%, *p* < 0.001, area under ROC curve 0.69). These findings are consistent with our results; however, the cardiovascular-related etiology of admission was observed in only 30% of Jansen's patients (compared with 100% of our patients). Lazzeri et al. [16] reported a significant impact of LC on prognosis only in patients in the worse hemodynamic state that were assessed on the basis of the Killip-Kimball classification and LVEF assessment; their results were also consistent with our findings. It is worth emphasizing that in contrary to the mentioned study our group consisted in nearly 60% of NSTEMI patients all of whom had invasive coronary angiography. The LC was significantly elevated (4.7 versus 3.7 mmol/l, *p* < 0.025) in patients with deteriorated systolic function of the left ventricle that we arbitrarily defined as an LVEF below 45% in the acute echocardiographic assessment prior to admission to cath lab. Vermeulen et al. [17] reported that an acute hemodynamic state in the patient (expressed by symptoms like increased heart rate, decreased blood pressure, and the presence of diabetes and distal embolization of coronary arteries) significantly impacted LC elevation and the 30-day mortality rate. These findings were also consistent with our results.

## 7. Conclusions

The point-of-care LC assessment of capillary blood using a handheld analyzer is a safe, easy, and cost-effective method to stratify the risk of late-onset heart failure and death after AMI. Its prognostic potential was conserved irrespective of clinical signs of shock.

## 8. Limitations of the Study

This is a pilot study; thus, the number of patients is relatively small. It was also a single-center study. However, the authors collecting the data did not interfere with the management process. Due to the design of the study and the national law regulations, all patients had to give informed and written consent; hence, the patients who were unconscious were excluded from the study. This could have impacted the potential selection bias and may have resulted in the absence of in-hospital deaths.

## Figures and Tables

**Table 1:** Patient characteristics according to the type of myocardial infarction.

	STEMI (*n* = 60)	NSTEMI (*n* = 85)	*p*
Age	59.85	62.86	ns
Women	21 (35%)	25 (30%)	ns
Hypertension	50 (83%)	76 (89%)	ns
Diabetes	20 (33%)	20 (24%)	ns
Smoking	36 (60%)	37 (44%)	ns
Atrial fibrillation	5 (8%)	6 (7%)	ns
Systolic BP (mmHg)	136	140	ns
Diastolic BP (mmHg)	81	83	ns
Shock	7 (12%)	1 (1%)	ns
IABP	2 (3%)	0 (0%)	ns
eGFR < 60 (ml/min/1.73^2^)	5 (8%)	11 (13%)	ns
Previous CABG	1 (2%)	3 (4%)	ns
Previous cardiac arrest	3 (5%)	2 (2%)	ns
GPIIb/IIIa inhibitors	29 (48%)	8 (9%)	*p* < 0.001
EDV (ml)	97	90	ns
LVEF (%)	42	49	*p* < 0.005
Hospitalization time (days)	8.8	8.0	ns
Heart rate (1/min)	78	82	ns
BMI	27.7	28.6	ns
Time from symptom onset to FMC (hours)	7.77 ± 7.19; 6.00 (3.25, 9.00)	22.04 ± 21.68; 12.00 (8.00, 24.00)	*p* < 0.001

Abbreviations: BMI: body mass index. BP: blood pressure. CABG: coronary artery bypass grafts. EDV: end-diastolic volume. eGFR: estimated glomerular filtration rate. GPIIb/IIIa: glycoprotein IIb/IIIa inhibitors. IABP: intra-aortic balloon pump. LVEF: left ventricular ejection fraction. NSTEMI: non-ST segment elevation myocardial infarction. STEMI: ST segment elevation myocardial infarction.

**Table 2:** Biochemical parameters in STEMI versus NSTEMI patients.

Parameter	STEMI (*n* = 60)	NSTEMI (*n* = 85)	*p*
Lactate concentration (mmol/l)	4.0	4.1	ns
Glycemia (mmol/l)	8.4	7.3	*p* < 0.001
Total cholesterol (mg/dl)	220	215	ns
Triglycerides (mg/dl)	126	124	ns
HDL (mg/dl)	60	55	ns
LDL (mg/dl)	135	136	ns
cTn (ng/l)	847	357	ns
CK-MB (IU/l)	64	49	ns
Creatinine (umol/l)	78	81	ns
RBC (10^6^/ul)	4.8	4.8	ns
WBC (10^3^/ul)	12	10	*p* < 0.025
PLT (10^3^/ul)	259	239	ns
HGB (g/dl)	14	14	ns

CK-MB: creatine kinase MB. cTn: cardiac troponin. HDL: high-density lipoproteins. HGB: hemoglobin. LDL: low-density lipoproteins. NSTEMI: non-ST elevation myocardial infarction. PLT: platelet count. RBC: red blood cell count. STEMI: ST elevation myocardial infarction.

**Table 3:** ROC-derived cut-off values of LC for each endpoint.

Endpoint during follow-up	LC (mmol/l)	Sensitivity and specificity (%)	AUC ± SD	95% CI
Death (I)	5.35	80	0.9 ± 0.05	(0.80–0.99)
Heart failure (II)	4.14	69	0.74 ± 0.07	(0.61–0.88)
Re-MI (III)	3.28	49	0.56 ± 0.07	(0.43–0.69)

AUC: area under the curve. CI: confidence interval. LC: lactate concentration. Re-MI: recurrent myocardial infarction. ROC: receiver operating characteristic. SD: standard deviation.

**Table 4:** Cox regression model for death occurrence in one year of follow-up.

Endpoint I	Univariate Cox regression analysis			Multivariate Cox regression analysis		
	HR	95% CI	*p*	HR	95% CI	*p*
HGB	0.68	(0.47–0.97)	0.030	—	—	—
LC	1.29	(1.11–1.51)	0.001	1.41	(1.13–1.76)	0.002
LDL	0.98	(0.97–0.99)	0.004	0.97	(0.95–0.99)	0.008
LM > 50%	7.16	(1.15–44.47)	0.031	11.06	(1.34–91.04)	0.023

HGB: hemoglobin. LC: lactate concentration. LDL: low-density lipoproteins. LM: left main coronary artery.

**Table 5:** Cox regression model for heart failure occurrence in one year of follow-up.

Endpoint II	Univariate Cox regression analysis			Multivariate Cox regression analysis		
	HR	95% CI	*p*	HR	95% CI	*p*
AF	3.53	(0.96–12.98)	0.05	—	—	—
BMI	1.12	(1.01–1.24)	0.03	1.20	(1.07–1.35)	0.002
CREAT	1.03	(1.01–1.04)	0.001	1.04	(1.02–1.06)	0.0004
GPIIb/IIIa	3.01	(1.03–8.78)	0.04	5.00	(1.52–16.42)	0.007
IABP	8.23	(1.03–66.06)	0.04	—	—	—
LC	1.17	(1.04–1.31)	0.01	1.21	(1.05–1.40)	0.007
LM > 50%	4.53	(1.39–14.82)	0.01	—	—	—
Diabetes	2.73	(0.94–7.96)	0.06	—	—	—
Shock	5.39	(1.46–19.88)	0.01	—	—	—
STEMI	2.59	(0.85–7.91)	0.09	—	—	—
WBC	1.12	(0.99–1.25)	0.06	—	—	—

AF: atrial fibrillation. BMI: body mass index. CREAT: creatinine. GPIIb/IIIa: glycoprotein IIb/IIIa inhibitors. IABP: intra-aortic balloon pump. LC: lactate concentration. LM: left main coronary artery. STEMI: ST elevation myocardial infarction. WBC: white blood cells.

**Table 6:** Cox regression model for recurrent myocardial infarction in one year of follow-up.

Endpoint III	Univariate Cox regression analysis			Multivariate Cox regression analysis		
	HR	95% CI	*p*	HR	95% CI	*p*
CREAT	1.02	(1.01–1.04)	0.00	1.02	(1.01–1.04)	0.001
DES	2.54	(0.88–7.37)	0.08	—	—	—
Glycemia	1.13	(1.03–1.25)	0.01	—	—	—
HDL	1.01	(1.00–1.02)	0.01	—	—	—
LAD > 50%	4.52	(1.00–20.37)	0.05	7.08	(1.11–44.97)	0.034
LVEF	0.96	(0.92–1.00)	0.03	—	—	—
Number of stents	1.79	(1.04–3.08)	0.03	1.69	(1.02–2.81)	0.037
Previous SCA	4.27	(0.95–19.26)	0.05	23.55	(3.25–170.86)	0.001
Smoking	0.20	(0.06–0.71)	0.01	0.14	(0.04–0.56)	0.005
Stent length	1.02	(1.00–1.04)	0.08	—	—	—

CREAT: creatinine. DES: drug-eluting stent. HDL: high-density lipoproteins. LAD: left anterior descending coronary artery. LVEF: left ventricular ejection fraction. SCA: sudden cardiac arrest.
